# 

**Published:** 1916-11

**Authors:** 


### Publishers' Announcement

ADVERTISING has done as much for the out-of-town districts and the country towns as railroads and traction lines have. It has lessened the distance between buyer and seller. Without advertising the buyers in different sections of the country would be isolated from the business districts in the great cities. This is but another synonym of our statement that advertising is not only of service to the seller of merchandise, but is of equally valuable service to the buyer. The distance between buyer and seller is eliminated by advertising as it makes known the saleable merchandise to the buyer, no matter how far away the factory or dealer is.

The DENTAL REGISTER, now in its seventieth year, is the pioneer of dental advertisers, and has helped many a dentist to select his chair and cabinet and sundry items in his office, without having to make a trip on a train or a trolley car to the dealer.

If you made the statement that you would not buy any advertised brands of merchandise, you would not need a bank account to pay for what you bought, for every brand of merchandise that is bought, you will find extensively advertised; or if you made the statement that you would not buy from a firm that advertises, you would certainly have to do without many items you wanted, for the firms that advertise are the very firms that have the goods you want.

### ANNOUNCEMENTS

INFORMATION FOR PERSONS DESIRING TO ENTER THE DENTAL CORPS OF THE UNITED STATES NAVY. A candidate for appointment to the Dental Corps of the Navy as dental surgeon must be a citizen of the United States, between 24 and 30 years of age, a graduate of a standard medical or dental college, trained in the several branches of dentistry, of good moral character, and of unquestionable professional repute.

The act of August 29, 1916, provides in part as follows:

"That the president of the United States is hereby authorized to appoint and commission, by and with the advice and consent of the Senate, dental surgeons in the Navy at the rate of one for each one thousand of the authorized enlisted strength of the Navy and Marine Corps, who shall constitute the Naval Dental Corps, and shall be a part of the Medical Department of the Navy. Original appointments to the Naval Dental Corps shall be probationary for a period of two years, and may be revoked at any time during the probationary period by the President: *Provided,* That the rank of such officers of the same date of appointment among themselves at the end of said probationary period shall be determined by the recommendations of an examining board appointed by the Secretary of the Navy, which board shall conduct a competitive examination, based upon both service record and professional attainments, in accordance with such regulations as may be prescribed by the Secretary of the Navy, and the rank of such officers so determined shall be as of date of original appointment with reference to other appointments to the naval service: *Provided further,* That all appointees to the grade of dental surgeon shall be citizens of the United States between twenty-four and thirty years of age, and shall be graduates of standard medical or dental colleges and trained in the several branches of dentistry, and who shall, before appointment, have successfully passed moral, physical, and professional examinations before medical and professional examining boards appointed by the Secretary of the Navy, and have been recommended for appointment by such boards.

"Dental surgeons shall have the rank, pay, and allowances of lieutenants (junior grade) until they shall have completed five years' service. Dental surgeons of more than five but less than twenty years' service shall, subject to such examinations as the Secretary of the Navy may prescribe, have the rank, pay, and allowances of lieutenant. Dental surgeons of more than twenty years' service shall, subject to such examinations as the Secretary of the Navy may prescribe, have the rank, pay, and allowances of lieutenant commander: *Provided,* That the total number of dental surgeons with the rank, pay, and allowances of lieutenant commander shall not at any time exceed ten."

Application for a permit to take the examination should be made to the Chief of the Bureau of Navigation, Washington, D. C., via the Surgeon General, United States Navy, and according to the form prescribed. This application must be in the handwriting of the candidate and must be accompanied by the following certificates:

(*a*) Letters or certificates from two or more persons of good repute, testifying from personal knowledge to good habits and moral character.

(*b*) A certificate to the effect that the applicant is a citizen of the United States.

(*c*) Certificate of preliminary education. The candidate must submit a certificate of graduation from an accepted high school or an acceptable equivalent.

(*d*) Certificate of dental education. This certificate should give the name of the school and the date of graduation.

(*e*) If the candidate has had special educational or professional advantages, certificates to this effect, signed by the proper authorities, should also be forwarded.

The applicant will save unnecessary correspondence if he will make sure when submitting his application that the qualifications enumerated above are clearly and plainly described in his letters or certificates.

FORM OF APPLICATION.

SURGEON GENERAL, United States Navy, Washington, D. C.:

Dear Sir: I request permission to be examined for an appointment as dental surgeon in the United States Navy. I was born at ———, and was — years of age on the — day of ———, 191—; am a citizen of the United States, residing in ———, county of ———, in the State of ——— I am a graduate of ——— dental (medical) school, in the State of ———, and was licensed to practice dentistry in the state of ——— in ———.

I forward herewith letters testifying to my moral character, habits, citizenship, preliminary education, professional education, and qualifications.

Very respectfully, ————————————

(Sign the name in full.)

THE CHIEF OF THE BUREAU OF NAVIGATION,

*Navy Department, Washington, D. C.* (via The Surgeon General, United States Navy).

A candidate whose preliminary qualifications appear to be satisfactory will receive a formal permit to present himself for examination.

THE EXAMINATION.

When a candidate presents himself for examination he must bring with him the testimonials as to character and professional fitness, diploma of preliminary and professional education, and a certificate that he is a citizen of the United States; those forwarded with his application being returned to him for this purpose.

The examination is conducted in the following order: I. Moral. II. Physical. III. Professional.

### II. PHYSICAL EXAMINATION.

The physical examination is thorough, and the candidate is required to certify on oath that he is free from all mental, physical, and constitutional defects. (Applicants are advised to carefully read the special circular regarding physical qualifications published herewith.)

Acuteness of vision, 12-20 for each eye, unaided by glasses, but capable of correction, by aid of lenses, to 20-20 is obligatory. Color perception must be normal and the teeth good.

If the candidate is found to be physically disqualified, his examination is concluded; if found to be physically qualified, his examination is continued as follows:

### III. PROFESSIONAL EXAMINATION.

1. Letter to the board describing in detail his general and professional education.

2. Theoretical (written and oral) in the following subjects: Anatomy, physiology, histology, physics, chemistry, metallurgy, dental materia medicia and therapeutics, dental pathology and bacteriology, orthodontia, oral surgery, operative dentistry (theory), and prosthetic dentistry (theory).

3. Practical (clinical) in operative dentistry and prosthetic dentistry.

4. An oral examination is conducted in the following subjects of preliminary education: Arithmetic, grammar, general history, physics, general literature, and Latin. Applicants holding diplomas or certificates from reputable literary or scientific colleges, normal schools, or high schools may submit such diplomas or certificates for the consideration of the board in this connection.

A successful candidate, upon completion of his examination, will be notified by the president of the board that he has been found qualified.

With the consent of the board, a candidate may withdraw at any period from further examination, and may at a future time present himself for re-examination. The board may conclude the examination (written, oral, and practical) at any time, and may deviate from this general plan as it may deem best for the interests of the naval service.

*No allowance will be made for the expense of persons appearing for examination.*

The tenure of office in the Dental Corps of the Navy is for life, unless sooner terminated by removal, resignation, disability, or other casualty.

All officers of the Dental Corps are retired from active service at the age of 64 years, and when so retired (or when retired from active service for disability or other casualty contracted in the line of duty before that age) receive an annual pay for life amounting to three-fourths of the pay of the grade or rank held by them at the time of retirement, including the increased pay allowed for length of service as explained below.

When an officer of the Navy, including dental officers, has been 30 years in the service, he may, upon his own application, in the discretion of the President, be retired from active service and placed upon the retired list at an annual pay for life amounting to three-fourths of the pay of the grade or rank held by him at the time of retirement, including the increased pay allowed for length of service.

Immediately upon official notification of the death from wounds or disease not the result of his own misconduct of any officer of the Navy, the Paymaster General of the Navy shall cause to be paid to the widow, and if no widow, to the children, and if there be no children, to any other dependent relative of such officer previously designated by him, an amount equal to six months' pay at the rate of pay received by such officer at the date of his death.

When traveling under orders by other than public conveyance, officers of the Navy, including dental officers, receive 8 cents a mile to defray the expenses of such travel performed from point to point within the United States, and when so traveling abroad are allowed actual personal expenses estimated on a liberal basis in accord with the position of the officer, both as regards admissible items of expense and the cost of such items.

For every five years' service the pay of officers is increased 10 per cent (though not to exceed 40 per cent), calculated on the annual base pay of their grade, as shown in the appended table.

When an officer goes to sea or leaves the continental limits of the United States under assignment to stations or for the performance of other duties beyond the seas, his pay is increased 10 per cent.

When two or more candidates are examined at the same time, their appointments will be in order of merit reported by the board.

Officers of the Dental Corps are entitled to all the military courtesies and consideration that go with their rank and are accorded to officers of other branches of the service in a similar grade. They wear the same uniform as other officers of the Navy, with a designating device distinctive of their corps.

PAY AND ALLOWANCE TABLE.

| Height. | Weight. | Chest (mean circumference). | Height. | Weight. | Chest (mean circumference). |
|---|---|---|---|---|---|
| *Inches.* | *Pounds.* | *Inches.* | *Inches.* | *Pounds.* | *Inches.* |
| 66 | 132 | 33½ | 70 | 155 | 35½ |
| 67 | 134 | 34 | 71 | 162 | 36 |
| 68 | 141 | 34½ | 72 | 169 | 36¼ |
| 69 | 148 | 34¾ | 73 | 176 | 36¾ |

For further information address the Surgeon General, United States Navy, Navy Department, Washington, D. C.

PHYSICAL QUALIFICATIONS REQUIRED OF PERSONS DESIRING COMMISSIONS IN THE NAVY AND MARINE CORPS.

1. The physical qualifications of applicants for appointment as officers in the Navy and Marine Corps are decided upon by an examining board consisting of medical officers of the Navy. The physical examination of candidates will, as a rule, precede the mental and professional. No one found physically disqualified will be examined further. No material physical defect will be waived in any case for any reason.

2. A candidate must declare under oath that he labors under no mental or constitutional disease or weakness, nor any other imperfection or disability which may interfere with the most efficient discharge of the duties of an officer in any climate.

3. Candidates for appointment must be between the following ages:

Dental Corps, dental surgeon.................................. 24 to 30
Dental Reserve Corps, assistant dental surgeon, for subsequent appointment as dental surgeon, Dental Corps.............. 24 to 29
Dental Reserve Corps, dental surgeon........................ 22 to 45

The candidate must be under thirty years of age when he completes the course of instruction at the Naval Dental School and is further examined to determine his fitness for commission in the Dental Corps.

Applicants will be required to satisfy the examining board regarding age.

4. For all corps herein mentioned, excepting officers for aeronautic duty only, the height must not be less than 5 feet 6 inches stripped.

TABLE OF PHYSICAL PROPORTIONS FOR HEIGHT, WEIGHT, AND CHEST MEASUREMENT.

| Length of service. | Pay per annum on shore. | Allowances per annum for quarters. | Total pay and allowances per annum on shore. | Pay per annum at sea. |
|---|---|---|---|---|
| Dental surgeons, rank of lieutenant (junior grade) ............. | $2,000 | $432 | $2,432 | $2,200 |
| Dental surgeons, rank of lieutenant | 2,400 | 576 | 2,976 | 2,640 |
| After 5 years in service...... | 2,640 | 576 | 3,216 | 2,904 |
| After 10 years in service...... | 2,880 | 576 | 3,456 | 3,168 |
| After 15 years in service...... | 3,120 | 576 | 3,696 | 3,432 |
| Dental surgeons, rank of lieutenant commander: | | | | |
| After 5 years in service........ | 3,300 | 720 | 4,020 | 3,630 |
| After 10 years in service........ | 3,600 | 720 | 4,320 | 3,960 |
| After 15 years in service........ | 3,900 | 720 | 4,620 | 4,200 |
| After 20 years in service........ | 4,000 | 720 | 4,720 | 4,400 |

It is not necessary that the applicant should conform exactly to the figures in the foregoing table, which is given to show what is regarded as a fair standard of physical proportions. A variation not exceeding 15 pounds, not to fall below 132 pounds in weight or 1 inch in the mean chest measurement, below the standard given in the table, is admissible when the candidate for appointment is active, has firm muscles, and is evidently vigorous and healthy. A chest expansion of less than 2½ inches is a sufficient cause for the rejection of the applicant.

Any one of the following conditions will be sufficient to cause rejection:

(*a*) Feeble constitution, poor physique, impaired general health.

(*b*) Any disease or deformity, either congenital or acquired, which would impair efficiency, such as weak or deranged intellect, cutaneous diseases, parasites of the skin or its appendages, deformity of the skull, abnormal curvature of the spine, torticollis, inefficiency of joints or limbs, deformity of joints or bones, either congenital or the result of disease or injury, epilepsy, or other convulsions, diseases of the eye, defective vision, color blindness, impaired hearing or disease of the ear, chronic nasal catarrh, ozena, polypi, great enlargement of the tonsils, impediment of speech, disease of heart or lungs, enlarged abdominal organs, evidence of sclerosis, tumors, hernia, undescended testicle, large varicocele, sarcocele, hydrocele, stricture, fistula, hemorrhoids, varicose veins, disease of the genito urinary organs, deformed or diseased feet, evidences of intemperance or of the morbid use of drugs, loss of many teeth or teeth generally unsound (teeth properly filled not to be considered unsound). Every applicant must have at least 20 sound teeth, and of these not less than 4 opposed incisors and 4 opposed molars.

(*c*) Any acute disease.

5. Acuteness of vision must be as follows:

For the Medical Corps, Dental Corps, and for chaplains, not less than 12-20 for each eye, unaided by glasses, and capable of correction by glasses to 20-20.

For acting ensigns for engineering duty and the Pay Corps, not less than 15-20 for each eye, unaided by glasses, and capable of correction by glasses to 20-20.

For the Marine Corps, 18-20 for each eye, unaided by glasses, and capable of correction by glasses to 20-20.

For the Medical Reserve Corps and Dental Reserve Corps, for active service only in time of emergency, the physical qualifications for candidates shall conform in general to those for the other corps of the Navy.

October, 1916.

---

ST. LOUIS DENTAL SOCIETY. The sixtieth anniversary meeting of the St. Louis Dental Society will be held at the Planters Hotel, St. Louis, November 16, 17, 18, 1916.

A program offering the latest information and a rational procedure in the management of the vital problems confronting the profession at this time will be presented by men of recognized authority on the subjects.

Dr. Weston A. Price, of the Research Institute of the National Dental Association, will lecture on "The Present Status of Our Knowledge of the Relation of Mouth Infections to Systemic Disease." This will include a review of the recent researches on focal infection compared to clinical data, the processes by which organisms attack tissues, and the principles of resistance to infection, illustrated with stereopticon and motion pictures.

Dr. C. N. Johnson, in a paper on "Some of the Present Tendencies in Operative Dentistry," will discuss the efficiency of prophylaxis in preventing disease. the influence of silicates on modern practice, and the proper attitude of the dentist, the physician, and the radiographer toward focal infection associated with teeth.

Dr. Elmer S. Best, who has standardized the "Technic of Pulp Canal Surgery," will give the differential indications for sterilization and filling, apicoectomy and curettage, and extraction. He will outline the complete technic of aseptic pulp canal operations, and give a clinical demonstration.

Dr. Edward T. Tinker will teach the "Technic of Bridge Abutments on Vital Teeth," showing the detail of construction and radiographic proof of the practicability of conserving pulps in bridge work.

Dr. W. E. Cummer, of Toronto, will offer a solution to the problem of supplying artificial substitutes in the semi-edentulous mouths of the martyrs to the indiscriminate extraction of pulpless teeth. Under the title of "Partial Dentures," the glorified descendant of the inefficient, unhygienic fixed bridge and "ordinary" partial denture will be described.

One afternoon will be devoted to general clinics, another to manufacturers' exhibits and clinics, and the third to group clinics by the members of the St. Louis Dental Society, with the following sections: Prophylaxis, pulp operations, inlays, gold and amalgam fillings, porcelain crowns, inlays and stains, shell jacket, and dowel crowns, bar and anchor dentures, impressions and occlusion, deciduous teeth, radiodontia and oral surgery. Individual and study club clinics are solicited by the Clinic Committee, Dr. E. P. Dameron, DeMenil Bldg., St. Louis, Chairman.

Registration and admission strictly by National Dental Association membership cards, and the opening session will begin at 10 a. m., November 16, without formal ceremonies.

The following hotels are conveniently located and reservations should be made in advance. Planters, $1.50 to $6.00; Jefferson, $1.50 to $4.00; American, $1.50 to $2.50; Maryland, $1.00 to $4.00.

AMERICAN INSTITUTE OF DENTAL TEACHERS. The next annual meeting of the American Institute of Dental Teachers will be held at Hotel Adelphi, Philadelphia, Pa., January 23, 24, 25, 1917.

A number of papers, reports and discussions relating especially to dental education will mark this meeting. All dental teachers are cordially invited to be present.

ABRAM HOFFMAN, *Secretary.*

529 Franklin Street, Buffalo, N. Y.

---

OHIO STATE DENTAL SOCIETY will hold its meeting this year in Dayton, Ohio December 5, 6 and 7. The program is not ready to announce in this issue but we are assured that it will be fully up to the standard of former meetings. The dentists of Dayton will insure everybody a good time. If interested write to Dr. F. R. Chapman, Columbus, Ohio for a program.

